# Treatment of irreparable rotator cuff tears with superior capsular reconstruction

**DOI:** 10.1186/s40634-021-00342-1

**Published:** 2021-03-27

**Authors:** Nobuyuki Yamamoto, Eiji Itoi

**Affiliations:** Department of Orthopaedic Surgery, Tohoku University School of Medicine, 1-1, Seiryo-machi, Aoba-ku, Sendai, 980-8574 Japan

**Keywords:** Superior capsular reconstruction, Facia lata, Autograft, Allograft

## Abstract

**Abstract:**

The treatment of irreparable rotator cuff tears with severe muscle atrophy and fatty infiltration remains a challenge, especially in young patients. Many surgical procedures for these tears have been reported. No one surgical treatment has proven to be an optimal solution. Recently, reconstruction of the superior capsule with an allograft or autograft has gained popularity. In this manuscript, we reviewed the biomechanical and clinical reports that have assessed superior capsular reconstruction and clarified the issues about the surgical techniques and indication which have been discussed recently. Reconstruction of the superior capsule has shown promising early results with good clinical outcomes. Biomechanical studies have suggested various mechanisms of this procedure. Although good clinical results and biomechanical data are available, more research is necessary to further define the surgical indications and improve the surgical outcomes of this procedure.

**Level of evidence:**

Level V.

## Introduction

The treatment of irreparable rotator cuff tears with severe muscle atrophy and fatty infiltration remains a challenge, especially in young patients. In the literature, surgical options for irreparable rotator cuff tears have included arthroscopic debridement, partial repair, margin convergence, patch graft, tendon transfer, and reverse total shoulder arthroplasty (RSA), etc. Arthroscopic debridement, partial repair, and margin convergence can provide pain relief but has limited benefit with respect to improved range of motion and muscle strength [1, 2]. The partial repair may result in recurrent tear, which has been found to be as high as 52% [1]. RSA has demonstrated satisfactory clinical outcomes in elderly patients, whereas results have not been as promising in younger patients. When RSA was performed in patients younger than 60 years, a failure rate was reported to be 25% at 3-year follow-up [25]. In addition, complications seen with this procedure increase when used in younger patients [19]. Thus, elderly patients with less activity can be candidates for these procedures. No one surgical treatment has proven to be an optimal solution in younger active patients.

**Fig. 1:**
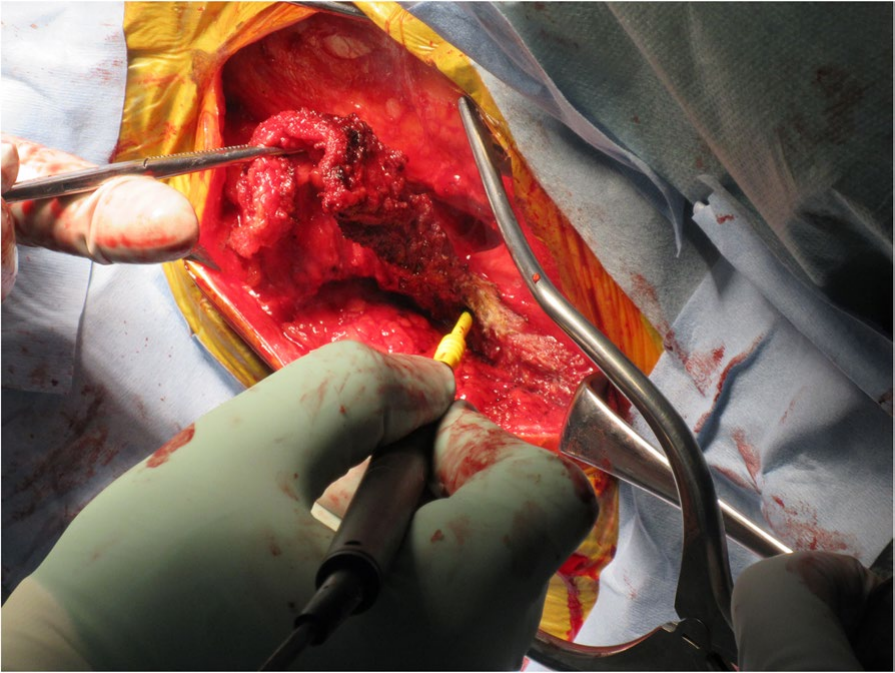
Harvesting the tensor fascia lata. The tensor fascia lata of the contralateral thigh was harvested in this patient

**Fig. 2:**
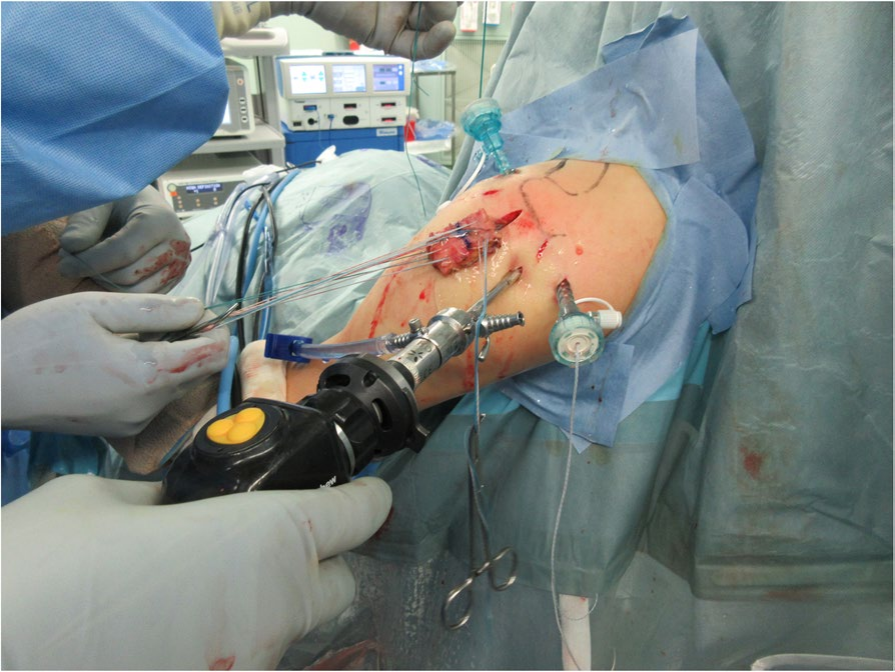
Graft insertion. The graft was inserted into the subacromial space through the enlarged lateral portal

**Fig. 3:**
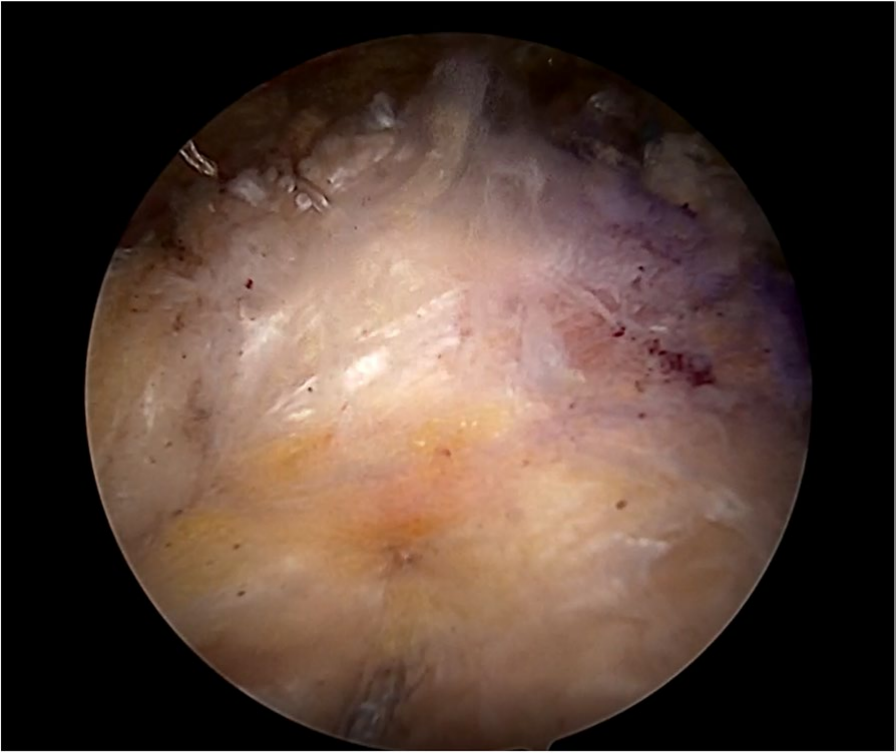
The tensor fascia lata autograft fixed. The lateral side of the fascia lata was attached to the rotator cuff footprint on the greater tuberosity by using the transosseous equivalent technique

Recently, superior capsular reconstruction (SCR) which was developed by a Japanese surgeon, Mihata et al. [9] has gained attention as a potential treatment for patients with irreparable rotator cuff tears (Figs. 1, 2 and 3). Subsequent early clinical results of SCR demonstrated excellent pain relief and an increase in shoulder function (range of motion and muscle strength) even in young patients [10]. They also reported that SCR restored shoulder function and resulted in high rates of return to recreational sports and physical work [15]. The 5-year follow-up study showed that healed SCR restored shoulder function [16].

The purpose of this study was to review the biomechanical and clinical reports that have assessed SCR and to clarify the issues about the surgical techniques and indications which have been discussed recently.

## Role of the superior capsule during motion

In the shoulder joint, the deepest layer of the rotator cuff is a thin continuous sheet of interwoven collagen fibrils, which is the capsule extending from the glenoid labrum medially to the humerus laterally [4]. The thickness of the capsule was demonstrated to be thicker than previously thought. An anatomical study [20] showed that the maximum capsular thickness was 9.1 mm at its attachment to the greater tuberosity between the infraspinatus and the teres minor. Accordingly, the attachment of the capsule occupied a substantial area of the greater tuberosity. Biomechanically, it has been demonstrated that the superior capsule acts as a static stabilizer to superior translation of the humeral head. Ishihara et al. [7] investigated the biomechanical contribution of the superior capsule. They found that the superior capsule played an important role in passive stability of the glenohumeral joint. In massive rotator cuff tears, the superior capsule is completely torn, which in turn results in the superior migration of the humeral head. Thus, it may be fair to say that SCR, which reconstructs the superior capsule, is a reasonable procedure to restore the superior stability.

## Evidence from biomechanical studies

There have been eight biomechanical studies investigating SCR and 5 of them were from the Mihata’s group, who developed SCR. Mihata et al. [9], in a cadaveric study, reported that the superior translation could be completely restored by using a patch graft to reconstruct the superior capsule. The effects of anterior and posterior continuity on shoulder biomechanics after SCR were also assessed [11]. They reported that SCR with side-to-side suturing restored the superior stability of the shoulder joint by establishing posterior continuity between the graft and residual infraspinatus tendon. The effect of the graft thickness and length was investigated in a biomechanical study using fresh cadavers [12]. Subacromial peak contact pressure and glenohumeral superior translation were measured under the three different SCR conditions: (1) a fascia lata allograft 4-mm thick and 15 mm longer than the distance from the superior glenoid to the greater tuberosity at 30º of glenohumeral abduction, (2) a fascia lata allograft 8-mm thick and with the same 15 mm relative length determined at 10º of glenohumeral abduction and (3) a fascia lata allograft 8-mm thick and with the 15-mm relative length determined at 30º of glenohumeral abduction. As a result, an 8-mm-thick graft of fascia lata had greater stability than did a 4-mm-thick graft. The effects of acromioplasty on biomechanics after SCR for irreparable supraspinatus tendon tears were assessed by Mihata et al. [13]. Glenohumeral superior translation and subacromial contact pressure were measured using fresh cadaveric shoulders. They concluded that adding acromioplasty decreased the subacromial contact area without increasing the subacromial contact pressure.

**Fig. 4:**
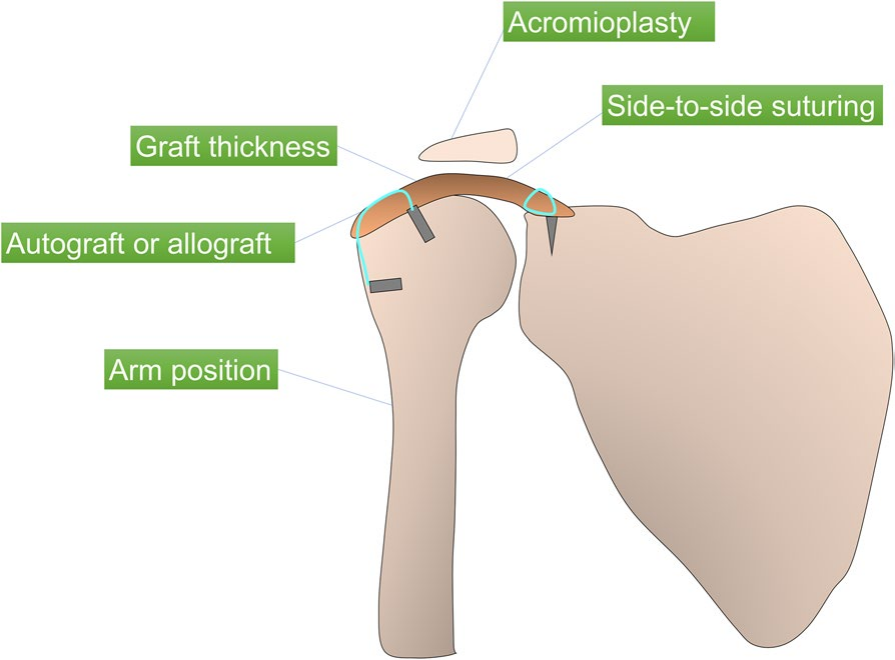
The biomechanics of superior capsular reconstruction. Diagram demonstrating the various effects of superior capsular reconstruction on shoulder biomechanics reported in the literature. The effects of graft thickness on subacromial contact pressure was assessed. Biomechanical cadaveric research has compared fascia lata allografts with human dermal allografts in superior capsular reconstruction. Subacromial contact pressure and superior translation were investigated in various arm positions. The effects of superior capsular reconstruction with and without acromioplasty on shoulder biomechanics was compared. The biomechanical role of side-to-side suturing of the graft during superior capsular reconstruction to the residual rotator cuff tendon was evaluated

Based on these biomechanical studies, there are two possible mechanisms how the SCR works. First, this procedure decreases superior humeral head migration and restores normal glenohumeral joint position just as the superior capsule does in live shoulders. Second, it is a spacer effect. The graft may work as a spacer to compress the humeral head against the acromion. As described below, Mihata et al. [10] proposed that the fascia lata autograft is folded to achieve a graft thickness of 6 to 8 mm to avoid re-tear after surgery. From the viewpoint of shoulder biomechanics, this thickness has another meaning: the graft works as a spacer. The subacromial balloon spacer which has been recently paid attention may provide the same mechanical effects to restore the humeral head position [26]. Although these biomechanical studies have shown the mechanisms of SCR, further studies are necessary to demonstrate the exact mechanism of this procedure (Fig. 4).

## Which is better, autograft or allograft?

In the first clinical reports of SCR by Mihata et al. [10], they used the fascia lata autograft. The fascia lata autograft is folded to achieve a graft thickness of at least 6 mm. The skin incision is usually more than 10 cm. Some patients complain thigh pain after surgery. Also, at least two surgeons are necessary to perform SCR, if the fascia lata is harvested parallel to the shoulder procedure. These have been pointed out to be disadvantages of SCR. As SCR has gain popularity mainly in United States, the use of dermal allografts was proposed due to several reasons [3]. First, we can eliminate the need for fascia lata autograft with a long skin incision. Second, operative time can be shortened because there is no need to harvest [5]. Third, the patients can avoid postoperative gait pain. Fourth, we can obtain the greater mechanical strength of the graft [14].

It seems that early clinical results using an allograft have been favorable. Denard et al. [5] reported arthroscopic SCR using dermal allograft provides a successful outcome in approximately 70% of cases. A biomechanical study [14] comparing the fascia lata allograft and dermal allograft showed that SCR with dermal allograft partially restored superior glenohumeral stability, whereas SCR with fascia lata allograft completely restored the superior glenohumeral stability. This might be due to the greater extensibility of the dermal allograft: it elongated by 15%. We need to wait to conclude that a dermal allograft can be used as a substitute for a fascia lata autograft until satisfactory clinical outcomes are reported from many institutes.

## Importance of graft thickness

When performing SCR using the fascia lata auto- or allograft, the surgeons need to know that the graft thickness is important to obtain the good clinical results. Mihata et al. [10] reported that a graft thickness of 6 to 8 mm should be achieved.

From the biomechanical viewpoint, this thickness has some meaning. Considering that the native shoulder capsule has been found to be 4.4 to 9.1 mm thick [20], a graft thickness of 6 to 8 mm in Mihata’s SCR is within this range. On the other hand, the maximal thickness of commercially available dermal allografts is 3 to 4 mm [5]. The effect of graft thickness on clinical outcomes is evident when separating clinical results by the graft thickness used. Denard et al. [4] clinically used the dermal allografts with the thickness ranging from 1 to 3 mm. The overall success rate was 67.8%, although 40% of the allografts were 1 mm thick. When SCR done with 1-mm grafts were excluded, the success rate increased to 75.5%. Another study by Hirahara et al. [6] demonstrated more significant improvements in pain and functional outcome measures in those with grafts > 3 mm. Thus, the ability of a thicker graft to act as a spacer to keep the humeral head down may explain the improved results. The difference between autograft and allograft outcomes may be due to the difference in thickness between grafts. In Mihata’s early study [10], 24 shoulders in 23 patients who had undergone SCR using autograft had improved outcome scores, with patients’ ASES scores improving from 24 to 93 at follow-up. In a multicenter prospective study [5], it was determined that SCR using a dermal allograft provided a successful outcome in approximately 68% of patients. More recently, Burkart et al. reported that 85% of grafts had fully healed, and 19% were judged to have unsatisfactory outcomes.

## Surgical technique of superior capsular reconstruction

Mihata et al. [10] fixed the graft to the superior glenoid with 2 suture anchors. Some surgeons [23] place 3 anchors (anterosuperior, superior, and posterosuperior) in the glenoid to provide complete coverage of the graft over the glenoid. Four anchors are usually used to secure the graft laterally to the humerus (in a linked bridging configuration). The original technique of SCR by Mihata [10, 15] was to fix the graft with the arm at 45°of abduction. Mihata et al. [13], in a biomechanical study, reported that SCR restored superior stability of the shoulder when the graft was fixed between 15° and 45° of shoulder abduction. Mihata et al. [12] evaluated the biomechanical role of side-to-side suturing of the graft during SCR. Superior translation was decreased when the SCR was repaired to the remaining posterior cuff but was not decreased in SCR alone. This study indicated that the graft should be sutured to the remnant posterior cuff tendon such as the infraspinatus or teres minor tendon.

## What are the indications and contraindications of SCR?

The overall indication for SCR varies among the surgeons and continues to change. As SCR is a relatively novel procedure, long-term outcome studies are not yet available in the literatures. At present, the best available evidence would suggest that SCR is a viable surgical option for young patients with massive, irreparable rotator cuff tears (typically involving supraspinatus and infraspinatus tendons) with severe muscle atrophy and fatty infiltration, who are not ideal candidates for RSA (Table 1). Looking at the literature, there have been many reports describing the surgical treatment for such young patients. However, few of them have been reported to bring satisfactory outcomes. Patients who had undergone a failed rotator cuff repair procedure and with minimal to no cuff tear arthropathy (Hamada Grade 1 or 2) are also candidates for SCR. Mihata et al. [17] reported that SCR reversed the pseudoparalysis in over 90% of the patients with mean active elevation of approximately 150°. As there are a couple of reports demonstrating that patients with pseudoparalysis are potential candidates for SCR [17, 27], we need further clinical studies to confirm if patients with pseudoparalysis are good indication of SCR. In early clinical report by Mihata et al. [10], all tears were large and massive including the supraspinatus and infraspinatus tendons. They reported excellent clinical outcomes. Also, Mihata et al. evaluated the biomechanical role of side-to-side suturing of the graft during SCR to the residual infraspinatous tendon. Superior translation was decreased when the SCR was repaired to the infraspinatous tendon but was not decreased in SCR alone. This biomechanical study indicated that the graft should be sutured to the remnant cuff tendon.

**Table 1:** Indication of the superior capsular reconstruction

	**Indication**
Age	Relatively young
Involved tendons	Supraspinatus and/or infraspinatus tendons
Subscapularis tendon	Intact or reparable
Muscle atrophy	Severe atrophy
Fatty infiltration	Goutallier 3 or 4
Cuff tear arthropathy	Hamada Grade 1 or 2
Pseudoparalysis	To be determied

In contrast, the contraindication for SCR are patients with severe cuff tear arthropathy (Hamada Grade ≥ 3) and those without a functional deltoid muscle due to axillary nerve palsy. In addition, since the reparability of the subscapularis was demonstrated to affect clinical outcomes after SCR [18], an irreparable subscapularis tendon tear is a contraindication for SCR. The indication of irreparable rotator cuff tears without severe cuff tear arthropathy in elderly patients for SCR should be discussed more. Further research is necessary to define the good indications and limitations of this procedure.

### Comparison to the clinical outcome of other surgical procedures

The treatment of irreparable rotator cuff tears with severe muscle atrophy and fatty infiltration remains a challenge, especially in young patients with high activity. Many reports have shown that RSA gets satisfactory clinical outcomes in elderly patients. However, results have not been as promising in younger patients. When RSA is performed in patients younger than 60 years, a failure rate is reported to be 25% at 3-year follow-up [25] and complications increase [19]. A surgical technique using a biodegradable subacromial balloon spacer implanted between the humeral head and acromion [22, 24] was introduced. This procedure is easy to perform and is less invasive. However, only the short-term results are available and it is still unclear whether or not we are able to obtain the same good clinical results in younger patients. Latissimus dorsi transfer has also been performed in patients with irreparable rotator cuff tears. Some investigators reported that latissimus dorsi transfer improved shoulder pain and function in physically active patients [8, 21]. However, these were retrospective studies and the number of the subjects was small. Thus, we still do not know whether young patients with high activity can be a candidate for these procedures. In a Mihata’s early report [10], SCR was performed in relatively young patients (mean age of 65 years) with excellent clinical results.

## Conclusions

Reconstruction of the superior capsule using fascia lata autograft has shown promising early results with improved clinical scores, range of motion, and muscle strength. Due to donor site morbidity, SCR using dermal allografts have gained popularity in United States. Biomechanical studies have suggested various mechanisms of this procedure supporting the good clinical outcomes. Despite the good clinical results and biomechanical data, further research is necessary to further define the indications, surgical technique, and limitations of SCR.

## References

[1] Berth A, Neumann W, Awiszus F, Pap G (2010). Massive rotator cuff tears: functional outcome after debridement or arthroscopic partial repair. J OrthopTraumatol.

[2] Burkhart SS (1997). Partial repair of massive rotator cuff tears: the evolution of a concept. OrthopClin North Am.

[3] Burkhart SS, Pranckun JJ, Hartzler RU (2020). Superior capsular reconstruction for the operatively irreparable rotator cuff tear: clinical outcomes are maintained 2 years after surgery. Arthroscopy.

[4] Clark JM, Harryman DT (1992). Tendons, ligaments, and capsule of the rotator cuff. Gross and microscopic anatomy. J Bone Joint Surg Am.

[5] Denard PJ, Brady PC, Adams CR, Tokish JM, Burkhart SS (2018). Preliminary results of arthroscopic superior capsule reconstruction with dermal allograft. Arthroscopy.

[6] Hirahara AM, Adams CR (2015). Arthroscopic superior capsular reconstruction for treatment of massive irreparable rotator cuff tears. Arthrosc Tech.

[7] Ishihara Y, Mihata T, Tamboli M, Nguyen L, Park KJ, McGarry MH, Takai S, Lee TQ (2014). Role of the superior shoulder capsule in passive stability of the glenohumeral joint. J Shoulder Elbow Surg.

[8] Lim TK, Bae KH (2019). Arthroscopic assisted latissimusdorsi tendon transfer for the management of irreparable rotator cuff tears in middle-aged physically active patients. Clin Shoulder Elb.

[9] Mihata T, McGarry MH, Pirolo JM, Kinoshita M, Lee TQ (2012). Superior capsule reconstruction to restore superior stability in irreparable rotator cuff tears: a biomechanical cadaveric study. Am J Sports Med.

[10] Mihata T, Lee TQ, Watanabe C, Fukunishi K, Ohue M, Tsujimura T (2013). Clinical results of arthroscopic superior capsule reconstructionfor irreparable rotator cuff tears. Arthroscopy.

[11] Mihata T, McGarry MH, Kahn T, Goldberg I, Neo M, Lee TQ (2016). Biomechanical role of capsular continuity in superior capsule reconstruction for irreparable tears of the supraspinatus tendon. Am J Sports Med.

[12] Mihata T, McGarry MH, Kahn T, Goldberg I, Neo M, Lee TQ (2016). Biomechanical effect of thickness and tension of fascia lata graft on glenohumeral stability for superior capsule reconstruction in irreparable supraspinatus tears. Arthroscopy.

[13] Mihata T, McGarry MH, Kahn T, Goldberg I, Neo M, Lee TQ (2016). Biomechanical effects of acromioplasty on superior capsule reconstruction for irreparable supraspinatus tendon tears. Am J Sports Med.

[14] Mihata T, Bui CNH, Akeda M, Cavagnaro MA, Kuenzler M, Peterson AB, McGarry MH, Itami Y, Limpisvasti O, Neo M, Lee TQ (2017). A biomechanical cadaveric study comparing superior capsule reconstruction using fascia lata allograft with human dermal allograft for irreparable rotator cuff tear. J Shoulder Elbow Surg.

[15] Mihata T, Lee TQ, Fukunishi K, Itami Y, Fujisawa Y, Kawakami T, Ohue M, Neo M (2018). Return to sports and physical work after arthroscopic superior capsule reconstruction among patients with irreparable rotator cuff tears. Am J Sports Med.

[16] Mihata T, Lee TQ, Hasegawa A, Fukunishi K, Kawakami T, Fujisawa Y, Ohue M, Neo M (2019). Five-year follow-up of arthroscopic superior capsule reconstruction for irreparable rotator cuff tears. J Bone Joint Surg Am.

[17] Mihata T, Lee TQ, Hasegawa A, Kawakami T, Fukunishi K, Fujisawa Y, Itami Y, Ohue M, Neo M (2018). Arthroscopic superior capsule reconstruction can eliminate pseudoparalysis in patients with irreparable rotator cuff tears. Am J Sports Med.

[18] Mihata T, Lee TQ, Hasegawa A, Fukunishi K, Kawakami T, Fujisawa Y, Ohue M, Doi M, Neo M (2020). Arthroscopic superior capsule reconstruction for irreparable rotator cuff tears: comparison of clinical outcomes with and without subscapularis tear. Am J Sports Med.

[19] Mulieri P, Dunning P, Klein S, Pupello D, Frankle M (2010). Reverse shoulder arthroplasty or the treatment of irreparable rotator cuff tear without glenohumeral arthritis. J Bone Joint Surg Am.

[20] Nimura A, Kato A, Yamaguchi K, Mochizuki T, Okawa A, Sugaya H, Akita K (2012). The superior capsule of the shoulder joint complements the insertion of the rotator cuff. J Shoulder Elbow Surg.

[21] Petriccioli D, Bertone C, Marchi G (2016). Recovery of active external rotation and elevation in young active men with irreparable posterosuperior rotator cuff tear using arthroscopically assisted latissimusdorsi transfer. J Shoulder Elbow Surg.

[22] Savarese E, Romeo R (2012). New solution for massive, irreparable rotator cuff tears: the subacromial “biodegradable spacer”. Arthrosc Tech.

[23] Schon JM, Katthagen JC, Dupre CN, Mitchell JJ, Turnbull TL, Adams CR, Denard PJ, Millett PJ (2017). Quantitative and computed tomography anatomic analysis of glenoid fixation for superior capsule reconstruction: a cadaveric study. Arthroscopy.

[24] Senekovic V, Poberaj B, Kovacic L, Mikek M, Adar E, Dekel A (2013). Prospective clinical study of a novel biodegradable sub-acromial spacer in treatment of massive irreparable rotator cuff tears. Eur J OrthopSurgTraumatol.

[25] Sershon RA, Van Thiel GS, Lin EC (2014). Clinical outcomes of reverse total shoulder arthroplasty in patients aged younger than 60 years. J Shoulder Elbow.

[26] Singh S, Reeves J, Langohr GDG, Johnson JA, Athwal GS (2019). The Subacromial balloon spacer versus superior capsular reconstruction in the treatment of irreparable rotator cuff tears: a biomechanical assessment. Arthroscopy.

[27] Takayama K, Yamada S, Kobori Y, Shiode H (2020). Association between the postoperative condition of the subscapularis tendon and clinical outcomes after superior capsular reconstruction using autologous tensor fascia lata in patients with pseudoparalytic shoulder. Am J Sports Med.

